# Undisclosed exposure to antiretrovirals prior to treatment initiation: An exploratory analysis

**DOI:** 10.4102/sajhivmed.v22i1.1200

**Published:** 2021-04-08

**Authors:** Lufuno G. Mavhandu-Ramarumo, Lisa A.M. Tambe, Nontokozo D. Matume, David Katerere, Pascal O. Bessong

**Affiliations:** 1 HIV/AIDS and Global Health Research Programme, Department of Microbiology, School of Mathematical and Natural Sciences, University of Venda, Thohoyandou, South Africa; 2 Department of Pharmaceutical Sciences, Faculty of Pharmaceutical Science, Tshwane University of Technology, Pretoria, South Africa; 3 Center for Global Health Equity, University of Virginia, Charlottesville, United States of America

**Keywords:** ART, pre-exposure, HIV drug resistance, adherence, exploratory analysis

## Abstract

**Background:**

The proportion of individuals with a history of exposure (‘pre-exposure’) to antiretrovirals (ARVs) prior to formal initiation into antiretroviral treatment (ART) is unknown.

**Objectives:**

This study describes the detection of ARVs in plasma and/or hair, of persons who self-reported no pre-exposure to ART at their first-time initiation onto ART in three clinics in the province of Limpopo, South Africa (SA).

**Method:**

Concentrations of tenofovir (TDF), emtricitabine (FTC) and efavirenz (EFV) in the plasma and hair of individuals initiating ART were analysed using a validated liquid chromatography tandem mass spectrometry (LC-MS/MS) method. Next generation sequences of HIV polymerase gene were analysed with Geneious software 11.15, and drug resistance (DR) mutations were determined according to the Stanford HIV Drug-Resistance database. Participants’ demographic data were collected on a structured questionnaire. Data that describe prior exposure to ARV were also collected by this self-reporting method.

**Results:**

Paired blood and hair samples were collected from 77 individuals newly initiated onto ART from 2017 to 2019. We detected at least one of the drugs in the plasma or hair of 41/77 (53.2%) patients who responded with a ‘no’ to the question ‘have you received ARVs before initiation onto ART?’ Thirty-one participants (*n* = 31/77, 40.3%) had TDF in either plasma or hair. Emtricitabine and EFV were found in the plasma or hair of 12/77 (15.6%) and 25/77 (32.4%) of participants respectively. Six (*n* = 6/77, 7.792%) had all three ARVs in plasma or hair. Prevalence of DR mutations at the > 5% significance threshold level in those known to have had ARV-exposure determined by LC-MS/MS prior to ART-initiation was not significant (χ^2^ = 0.798; *p* = 0.372), when compared to those who had no prior exposure but still showed DR.

**Conclusion:**

Antiretroviral levels in the hair of individuals initiating treatment imply prolonged prior-exposure to that ARV. The presence of ARV in plasma and hair of persons living with HIV (PLWH) who deny ARV-use, requires an explanation. A larger study at multiple sites and regular DR surveillance of ART-naïve PLWH will be necessary to confirm the generalisability of these findings to the wider South African population.

## Introduction

Considering evidence that treatment significantly reduces HIV transmission at the population level,^1^ the South African National Department of Health (SANDoH) introduced a Universal Test and Treat (UTT) programme in September 2016; a move whereby all tested persons enter treatment irrespective of the initiating level of the CD4+ T-cell count.^2^

Several key assumptions are included in UTT. These include the fact that the tested person ‘newly’ diagnosed with HIV is naïve to antiretroviral therapy (ART) and is infected with a strain of HIV susceptible to the recommended first-line ART regimen. Further, it is assumed that the prevalence of circulating drug-resistant virus in the pre-treated population is negligible and will not impact treatment outcomes. The World Health Organization (WHO) categorises the pre-treatment -drug-resistance at the population level as low if the incidence of drug resistance < 5%; moderate if 5% – 15% and high if > 15%. In South Africa (SA), the level of drug resistance in the pre-treated population has increased over time but is heterogeneous across and within provinces. At least one pre-treated population with a moderate level of resistance has been reported in each province, except the Northern Cape.^3^ It is essential that the proportion of patients with prior exposure to ART in one locality, but who re-initiate in another without disclosing previous ART-use, be kept small. Non-disclosure has been reported in SA and elsewhere in Africa, Asia and in South America^4,5^ and carries the risk of treatment failure with subsequent antiretroviral (ARV) regimens. Non-disclosure frequently accompanies ART default and re-initiation. A ±42% probability of this has been noted in a SA study and linked to the female gender, the duration of elapsed-time since defaulting and age.^6^ Non-disclosure may also occur following ‘forgotten’ pre-exposure and post-exposure ARV prophylaxis.

Another important UTT assumption is that recipients of ART will be adherent to the treatment. Poor adherence leads to inadequate viral suppression, the development of drug-resistance, treatment-failure and an increased risk of death.^7^ A 2019 study conducted amongst pregnant South African women attributed most virological failure (90%) to non-adherence.^8^ Non-adherence has also been associated with male gender, presentation at an advanced stage of HIV and with late/delayed access of ART.^9^ We have previously shown that adherence of more than 95% is required to achieve persistent viral load (VL) suppression.^10^

In this study we explore the relationship of prior ARV exposure to subsequent resistance on reinitiating treatment by analysing the plasma and/or hair samples of individuals at baseline, and at 6 months post-initiation for the presence of drug-resistant virus and to confirm non-adherence.

## Methodology

### Study setting and participants

This was an exploratory study, involving the use of available plasma samples collected prior to initiation of treatment (baseline) and at 6 months post-initiation amongst persons living with HIV (PLWH) who were participants in an unpublished parent HIV drug resistance and treatment outcome study. Both at ART initiation and at the 6-month post-initiation visit, strands of hair were cut approximately 3 mm away from the scalp at the back of each participant’s head, and stored in a sterile container for subsequent analysis. Matched plasma samples were taken at the same time. Study participants were recruited from three HIV-screening and ART-initiating sites in the province of Limpopo, SA namely, the University of Venda Campus Health Clinic in Thohoyandou, the Rethabile Community Health Centre in Polokwane and the Seshego Community Clinic in Seshego. Participants provided informed consent before recruitment into the study. All participants were adults, aged ≥ 18 years and were reportedly taking ART for the first time. No additional inclusion or exclusion criteria were applied. All participants were started on a fixed-dose combination (FDC) of tenofovir (TDF) + emtricitabine (FTC) + efavirenz (EFV): TEE. All demographic data including sex and age were collected by means of a structured questionnaire. Data on prior ARV drug exposure relied on self-reporting by participants.

### Measurement of CD4+ cell count and viral load

CD4+ cell count and VL measurement of study participants is shown in Appendix 1. Controls’ plasma and hair samples uninfected by HIV were used in the estimation of drug concentrations.

### Genome sequencing and the determination of viral resistance

Nucleoside/tide variant frequencies coding for drug resistance mutations were evaluated at both majority (> 20%) and minority (> 5% and > 1%) thresholds, using the find variation/single nucleotide polymorphisms feature from the annotation and prediction menu in Geneious,^11^ and analysed for drug resistance using the Calibrated Population Resistance protocol in the Stanford HIV Drug Resistance Database (see Appendix 1).

### Antiretroviral drug measurement/quantification in hair and plasma

Liquid chromatography-tandem mass spectrometry (LC-MS/MS) was used to quantify the individual components of TEE in the samples of hair and plasma, and was conducted at FARMOVS Bioanalytical Services Division, the University of the Free State, SA, a South African National Accreditation System accredited laboratory.

### Calibration standards, quality controls and test sample preparation for plasma and hair matrices

For the plasma preparation, the run comprised eight standard (STD) levels over the range of 20.00 ng/mL – 2560 ng/mL, with six levels of quality control (QC) samples extending over this range. Two replicates were included per level for each STD and each QC sample. One blank sample and one zero sample were included in the sample run.

The hair preparation run comprised eight STD levels over the ranges of 0.158 ng/mL – 20 ng/mL for EFV, 0.118 ng/mL – 15 ng/mL for FTC and 0.063 ng/mL – 8 ng/mL for TDF, with three levels of QC samples extending over this range. Two replicates were included per level for each STD and each QC sample. The STDs and QCs were interspersed amongst the study samples in a predetermined manner, with one blank sample and one zero sample included in the study sample run. See the detailed methodology for both matrices in Appendix 1.

### Data analysis

Data including the participants’ age, CD4 and HIV-RNA VL were calculated and reported as medians and interquartile ranges (IQR). The Spearman’s correlation coefficient (*R*^2^) was used to determine the response to treatment by comparing baseline and post-treatment CD4 and HIV RNA data. Results generated by the LC-MS/MS run were calculated using Watson LIMS™ software. The drug concentrations of TDF, FTC or EFV in plasma or hair of ARV-unexposed participants should be zero. The detection at baseline of these ARVs confirmed prior exposure. Data from the literature of adherent PLWH on ARVs provided plasma drug-concentrations for comparison. Observed plasma concentrations in the range of 13 ng/mL – 397 ng/mL, 11 ng/mL – 5000 ng/mL and 1000 ng/mL – 4000 ng/mL for TDF, FTC and EFV, respectively were considered adherent.^12,13^ The expected range of drug concentrations in hair ranged from 0.002 to 0.4 ng/mg, 0.02 ng/mg to 4 ng/mg and 0.05 ng/mg to 20 ng/mg for TDF, FTC and EFV, respectively.^14,15,16^ Drug concentrations at 6 months in plasma or hair that indicated adherence to treatment were analysed for associations with CD4+ cell count and VL levels. Next generation sequences of the HIV polymerase gene were analysed with Geneious software 11.15. Drug resistance (DR) mutations were determined according to the Stanford HIV Drug-Resistance database.

### Ethical considerations

The study protocol was approved by the University’s Human and Clinical Trial Research Ethics Committee (SMN/17/MBY/05/2106), and permission to access public sector health facilities was granted by the National Health Research Ethics Committee (NHREC) and obtained from the Provincial Department of Health of South Africa, and the relevant district managers. Signed informed consent was obtained from all participants prior to collection of specimens and demographic data. Confidentiality and anonymity were maintained.

## Results

### Demographic and clinical marker profiles of the study cohort

Paired blood and hair samples were collected from 77 PLWH newly started on ART between 2017 and 2019. *N* = 69/77 (89.6%) were females. The median age was 35 years, IQR, 27.25–42. The median baseline CD4+ cell count was 259 cells/µL, IQR, 137–382, and the median HIV-1 RNA VL level = 25 150 copies/mL, IQR, 64 275–84 514 (Table 1).

**TABLE 1:** Characteristics of the study cohort.

Parameters	Values
Number of patients	77
Median age, IQR	35 (27.25–42)
**Gender**	
Females (%)	69 (89.60%)
Males (%)	8 (10.40%)
Median viral load copies/mL (IQR) at baseline	25 150 (6427.5–84 514)
Median viral load copies/mL (IQR) post 6 months	38 (30–54.50)
Median CD4 counts cells/μL (IQR) at baseline	259 (137–382)
Median CD4 counts cells/μL (IQR) post 6 months	572 (347–781)

IQR, interquartile range; CD4, cluster of differentiation 4.

### Detection of antiretrovirals (ARVs) in the plasma or hair prior to treatment initiation at recruitment facilities

Considering the presence of TDF, FTC or EFV in the plasma or hair of participants, 41/77 (53.2%) were found to have been exposed to ARVs prior to treatment initiation. Thirty-four (*n* = 34) were females with a median age of 32.5 years (IQR 25.25–42) of whom 28/34 (80%), were aged < 45 years. Although the study included only eight males, seven (87.5%) were found to have prior exposure to ARVs. Their median age was 32 years, IQR 29.5–39.5. Table 2 provides the details of the individual ARVs and concentrations detected in the 41 participants.

**TABLE 2:** Characteristics of participants with at least one antiretroviral drug detected in hair or plasma prior to treatment initiation.

No	Sample code	Sex	Age	Drug concentrations (ng/mg) in hair at baseline			Drug concentrations (ng/mL) in plasma at baseline		
				TDF	FTC	EFV	TDF	FTC	EFV
1	AHDR-R280	F	33	0.028	00.000	00.000	00.000	00.000	00.000
2	AHDR-R283	M	32	0.330	00.000	00.000	00.000	00.000	00.000
3	AHDR-R287	M	31	0.027	0.300	1.070	00.000	00.000	00.000
4	AHDR-R289	F	40	00.000	00.000	0.068	00.000	00.000	00.000
5	AHDR-R292	F	25	00.000	00.000	0.096	00.000	00.000	00.000
6	AHDR-R295	F	48	00.000	0.863	17.432	00.000	00.000	1183.000
7	AHDR-R296	F	36	0.025	00.000	00.000	19.570	00.000	00.000
8	AHDR-R297	F	55	0.058	0.497	9.000	59.450	26.570	1051.000
9	AHDR-R298	F	23	00.000	00.000	00.000	4.356	00.000	00.000
10	AHDR-R299	F	37	00.000	00.000	0.079	00.000	00.000	00.000
11	AHDR-R302	F	65	0.490	1.300	13.230	51.110	114.500	895.800
12	AHDR-R306	F	32	0.025	00.000	00.000	00.000	00.000	00.000
13	AHDR-R307	M	40	0.043	00.000	0.880	00.000	00.000	00.000
14	AHDR-R311	F	42	00.000	00.000	0.138	00.000	00.000	00.000
15	AHDR-R315	F	55	00.000	00.000	0.257	00.000	00.000	00.000
16	AHDR-R319	F	37	00.000	00.000	0.075	15.700	00.000	00.000
17	AHDR-R320	F	52	00.000	00.000	00.000	61.880	00.000	00.000
18	AHDR-R322	F	57	0.027	0.201	16.700	43.890	139.100	1651.000
19	AHDR-R326	F	23	0.208	0.930	5.900	11.670	00.000	238.300
20	AHDR-R329	F	31	0.090	0.770	0.290	00.000	00.000	00.000
21	AHDR-R332	M	43	0.032	00.000	00.000	00.000	00.000	00.000
22	AHDR-R337	F	42	0.088	00.000	2.400	00.000	00.000	00.000
23	AHDR-R338	F	28	0.030	00.000	0.190	00.000	00.000	00.000
24	AHDR-R339	F	31	00.000	00.000	0.330	00.000	00.000	00.000
25	AHDR-R346	F	32	00.000	00.000	1.200	Not done	Not done	Not done
26	AHDR-R347	F	48	00.000	00.000	00.000	14.160	00.000	00.000
27	AHDR-R348	F	25	0.038	00.000	00.000	18.030	00.000	00.000
28	THDR-R26	F	22	00.000	00.000	0.170	Not done	Not done	Not done
29	AHDR-S083	M	28	00.000	0.230	00.000	25.530	00.000	00.000
30	AHDR-S087	F	30	00.000	00.000	00.000	35.250	00.000	00.000
31	AHDR-S090	F	39	00.000	00.000	00.000	36.430	00.000	00.000
32	AHDR-S091	F	35	00.000	00.000	00.000	23.060	00.000	00.000
33	AHDR-S094	F	53	00.000	00.000	0.267	35.330	290.600	769.900
34	AHDR-S095	F	23	00.000	00.000	0.071	28.820	00.000	00.000
35	AHDR-S096	M	39	00.000	00.000	00.000	52.860	00.000	00.000
36	AHDR-S099	F	26	00.000	00.000	0.110	25.030	00.000	00.000
37	AHDR-S103	F	22	00.000	00.000	0.180	223.300	13.100	00.000
38	AHDR-S105	F	21	00.000	00.000	0.104	00.000	00.000	00.000
39	AHDR-S112	F	27	00.000	0.210	2.670	13.030	00.000	9.500
40	AHDR-U01	F	22	00.000	00.000	00.000	27.310	00.000	00.000
41	AHDR-U02	M	20	00.000	0.270	8.000	31.310	00.000	00.000

TDF, tenofovir; FTC, emtricitabine; EFV, efavirenz.

Emtricitabine and EFV were observed in the plasma or hair of 12/77 (15.6%) and 25/77 (32.4%) of participants, respectively. Six (*n* = 6/77, 7.8%) had all three drugs (TDF/FTC/EFV) in plasma or hair.

### Detection of baseline drug resistance at time of treatment initiation

Baseline drug resistance/mutation data were available for 13 of the 41 participants (*n* = 13/41, 31.7%) in whom any of TDF, FTC or EFV, was detected in plasma or hair before the start of ART. Eight (*n* = 8/13, 61.5%) participants had at least one DRM. The following mutations were present at respective threshold levels of > 20%, > 5% and > 1%: K103N only (*n* = 1/13, 7.7%); K103N, and V106A (*n* = 3/13, 23.1%); K65R, K219E, K103N, V106A, N88D and I50V (*n* = 8/13, 61.5%). Details of the distribution of these mutations are given in Table 3.

**TABLE 3:** Participants having at least one drug at baseline in either the hair or plasma matrix with their drug resistance profiles.

No	Sample code	Sex	Drug resistance profiles			Drugs detected in hair at baseline			Drugs detected in plasma at baseline		
			DRM at > 20% threshold	DRM at > 5% threshold	DRM at > 1% threshold	TDF	FTC	EFV	TDF	FTC	EFV
1	AHDR-R 289	F	NO DRM	NO DRM	K65R (NRTI)	0.028	00.000	00.000	00.000	00.000	00.000
2	AHDR-R 297	F	NO DRM	K103N (NNRTI)	K103N (NNRTI)	0.058	0.497	9.000	59.450	26.570	1051.000
3	AHDR-R 299	F	NO DRM	K103N (NNRTI)	K103N (NNRTI)	00.000	00.000	0.079	00.000	00.000	00.000
4	AHDR-R 302	F	NO DRM	NO DRM	NO DRM	0.490	1.300	13.230	51.110	114.500	895.800
5	AHDR-R 307	M	NO DRM	NO DRM	NO DRM	0.043	00.000	0.880	00.000	00.000	00.000
6	AHDR-R 319	F	NO DRM	NO DRM	N88D (PI)	00.000	00.000	0.075	15.700	00.000	00.000
7	AHDR-R 339	F	NO DRM	NO DRM	K65R (NRTI)	00.000	00.000	0.330	00.000	00.000	00.000
8	AHDR-R 347	F	NO DRM	NO DRM	NO DRM	00.000	00.000	00.000	14.160	00.000	00.000
9	AHDR-R 348	F	K103N (NNRTI)	K103N, V106A (NNRTI)	K65R, K219E (NRTI), K103N, V106A (NNRTI)	0.038	00.000	00.000	18.030	00.000	00.000
10	AHDR-S 94	F	NO DRM	NO DRM	NO DRM	00.000	00.000	0.267	35.330	290.600	769.900
11	AHDR-S 95	F	NO DRM	NO DRM	NO DRM	00.000	00.000	0.071	28.820	00.000	00.000
12	AHDR-S 103	F	NO DRM	NO DRM	K65R (NRTI)	00.000	00.000	0.180	223.300	13.100	00.000
13	AHDR-S 112	F	NO DRM	NO DRM	I50V (PI)	00.000	0.210	2.670	13.030	00.000	9.500

DRM, drug-resistant mutation; NRTI, nucleoside reverse transcriptase inhibitors; NNRTI, non-nucleoside reverse transcriptase inhibitor; PI, protease inhibitor; TDF, tenofovir; FTC, emtricitabine; EFV, efavirenz.

We then looked at the resistance profiles of those, 36 of 77 (46.8%), who did not have ARVs in their plasma or hair. Data for this assessment were available for 18/36 (50%) participants. Seven participants 7/18 (38.9 %) had no DRMs. Of the 11 participants (61%) who harboured resistance mutations, the distribution was as follows: K65R (5.5%); D67N (5.6%); K65R, D67G, Y181C, G190E, V82A, I84V were observed in 11/18 (61%) participants at the > 20%, > 5% and > 1% threshold levels, respectively. Details of the distribution of these mutations are shown in Table 4.

**TABLE 4:** Participants with no drug at baseline in either the hair or plasma matrix with their drug resistance profiles.

No	Sample code	Sex	Drug resistance profiles			Drugs detected in hair at baseline			Drugs detected in plasma at baseline		
			DRM at > 20% threshold	DRM at > 5% threshold	DRM at > 1% threshold	TDF	FTC	EFV	TDF	FTC	EFV
1	AHDR-R294	F	NO DRM	NO DRM	NO DRM	00.000	00.000	00.000	00.000	00.000	00.000
2	AHDR-R300	F	NO DRM	NO DRM	K65R (NRTI), V82A (PI)	00.000	00.000	00.000	00.000	00.000	00.000
3	AHDR-R303	F	K65R (NRTI)	NO DRM	Y181C (NNRTI)	00.000	00.000	00.000	00.000	00.000	00.000
4	AHDR-R305	F	NO DRM	NO DRM	NO DRM	00.000	00.000	00.000	00.000	00.000	00.000
5	AHDR-R308	F	NO DRM	D67N (NRTI)	NO DRM	00.000	00.000	00.000	00.000	00.000	00.000
6	AHDR-R313	F	NO DRM	NO DRM	K65R (NRTI)	00.000	00.000	00.000	00.000	00.000	00.000
7	AHDR-R324	F	NO DRM	NO DRM	NO DRM	00.000	00.000	00.000	00.000	00.000	00.000
8	AHDR-R330	F	NO DRM	NO DRM	NO DRM	00.000	00.000	00.000	00.000	00.000	00.000
9	AHDR-R331	F	NO DRM	NO DRM	NO DRM	00.000	00.000	00.000	00.000	00.000	00.000
10	AHDR-R335	F	NO DRM	NO DRM	I84V (PI)	00.000	00.000	00.000	00.000	00.000	00.000
11	AHDR-R340	F	NO DRM	NO DRM	D67G (NRTI)	00.000	00.000	00.000	00.000	00.000	00.000
12	AHDR-R342	F	NO DRM	NO DRM	K65R(NRTI),G190E (NNRTI)	00.000	00.000	00.000	00.000	00.000	00.000
13	AHDR-R343	F	NO DRM	NO DRM	K65R (NRTI)	00.000	00.000	00.000	00.000	00.000	00.000
14	AHDR-R344	F	NO DRM	NO DRM	K65R (NRTI), V82A (PI)	00.000	00.000	00.000	00.000	00.000	00.000
15	AHDR-S088	F	NO DRM	NO DRM	NO DRM	00.000	00.000	00.000	00.000	00.000	00.000
16	AHDR-S102	F	NO DRM	NO DRM	NO DRM	00.000	00.000	00.000	00.000	00.000	00.000
17	AHDR-S110	F	NO DRM	NO DRM	K65R (NRTI)	00.000	00.000	00.000	00.000	00.000	00.000
18	AHDR-S111	F	NO DRM	NO DRM	K65R (NRTI)	00.000	00.000	00.000	00.000	00.000	00.000

DRM, drug-resistant mutation; NRTI, nucleoside reverse transcriptase inhibitors; NNRTI, non-nucleoside reverse transcriptase inhibitor; PI, protease inhibitor; TDF, tenofovir; FTC, emtricitabine; EFV, efavirenz.

More DRMs were observed in participants who had prior exposure to ARV drugs compared to those who did not, although this difference was not significant (χ^2^ = 0.798; *p* = 0.372). In both groups, nucleoside reverse transcriptase inhibitor (NRTI)-associated resistance mutations were the most prevalent, regardless of thresholds.

### Adherence at 6 months post-treatment initiation

We looked at the adherence profile at 6 months post-treatment initiation. Of the 77 participants who were recruited at initiation, paired plasma and hair samples were available for only 21, at the 6 month assessment. The adherence-analysis using plasma and hair levels of TDF/FTC/EFV, was based on the 21 participants. The concentrations of these ARVs administered as a FDC tablet, were within an acceptable adherence-range level for all 21 at their 6 month assessment. This observation is supported by a significant increase in the median CD4 = 572 (IQR 347–781) cells/µL at 6 months after starting treatment, compared to the median ART-initiating CD4 count of 259 (137–382) cells/µL (*R*^2^ = 0.560, *p* = 0.023). A significant decline in VL was observed at 6 months post-treatment initiation (20 RNA copies/mL – 20 RNA copies/mL), compared to values at treatment initiation (64275 RNA copies/mL – 84514 RNA copies/mL) (*R*^2^ = 0.48, *p* = 0.027). Details are shown in Table 5.

**TABLE 5:** Study participants’ adherence profiles after 6 months of antiretroviral therapy.

No	Sample code	Age	Sex	CD4 at treatment initiation (cells/µL)	CD4 6 months post-treatment (cells/µL)	Viral load at treatment initiation (copies/mL)	Viral load 6 months post-treatment (copies/mL)	Drugs detected at baseline						Drugs detected 6 months post-treatment					
								Hair			Plasma			Hair			Plasma		
								TDF	FTC	EFV	TDF	FTC	EFV	TDF	FTC	EFV	TDF	FTC	EFV
1	AHDR-R 295	48	F	547	672	207	20	00.000	0.863	17.432	00.000	00.000	1183.000	0000.000	9.958	16.298	61.490	139.700	287.300
2	AHDR-R 298	23	F	497	Not done	6950	20	00.000	00.000	00.000	4.356	00.000	00.000	0.124	0.019	12.026	58.750	182.500	2071.000
3	AHDR-R 305	31	F	664	Not done	31100	20	00.000	00.000	00.000	00.000	00.000	00.000	0.053	4.205	22.200	95.970	337.800	3738.000
4	AHDR-R 311	42	F	Not done	Not done	137000	22	00.000	00.000	0.138	00.000	00.000	00.000	0.138	2.027	27.166	108.700	352.200	3330.000
5	AHDR-R 315	55	F	416	Not done	919000	Not done	00.000	00.000	0.257	00.000	00.000	00.000	0.060	3.981	22.062	189.900	228.000	2419.000
6	AHDR-R 318	39	F	113	Not done	2600000	38	00.000	00.000	00.000	00.000	00.000	00.000	0.124	1.659	9.873	72.850	456.000	3562.000
7	AHDR-R 328	36	F	484	786	Not done	20	00.000	00.000	00.000	00.000	00.000	00.000	0.055	0.656	6.969	108.500	287.400	3450.000
8	AHDR-R 331	35	F	345	565	Not done	20	00.000	00.000	00.000	00.000	00.000	00.000	0.088	1.165	10.843	83.000	304.600	1283.000
9	AHDR-R 338	28	F	1007	1196	Not done	39.36	0.030	00.000	0.190	00.000	00.000	00.000	0.024	0.670	10.900	35.720	104.900	1134.000
10	AHDR-R 340	36	F	278	592	Not done	48	00.000	00.000	00.000	00.000	00.000	00.000	0.065	2.243	6.574	66.180	194.200	1688.000
11	AHDR-S 88	31	F	191	142	16900	20	00.000	00.000	00.000	00.000	00.000	00.000	0.087	4.563	29.440	73.200	418.900	6685.000
12	AHDR-S 92	46	F	164	Not done	3390	20	00.000	00.000	00.000	00.000	00.000	00.000	0.077	3.531	10.926	69.100	229.800	2671.000
13	AHDR-S 94	53	F	101	347	100000	20	00.000	00.000	0.267	35.330	290.600	769.900	0.039	1.201	9.953	121.400	353.100	1944.000
14	AHDR-S 101	30	M	500	572	9120	20	00.000	00.000	00.000	00.000	00.000	00.000	0.053	0.152	13.088	0000.000	18.480	506.800
15	AHDR-S 102	43	F	567	781	2746469	20	00.000	00.000	00.000	00.000	00.000	00.000	0.256	3.336	30.200	95.000	290.400	3661.000
16	AHDR-S 106	33	F	334	Not done	68941	71	00.000	00.000	00.000	00.000	00.000	00.000	0.061	2.536	30.789	82.700	352.900	2999.000
17	AHDR-S 110	29	F	74	Not done	17600	20	00.000	00.000	00.000	00.000	00.000	00.000	0.031	10.359	34.847	197.300	530.300	00.000
18	AHDR-S 111	35	F	Not done	Not done	6400	20	00.000	00.000	00.000	00.000	00.000	00.000	0.408	2.034	1.430	57.820	265.400	1180.000
19	AHDR-S 113	54	F	Not done	1148	5490	20	00.000	00.000	00.000	00.000	00.000	00.000	0.161	8.591	29.272	96.610	344.000	2781.000
20	AHDR-S 117	41	F	85	261	84600	20	00.000	00.000	00.000	00.000	00.000	00.000	0.051	4.557	36.880	117.800	229.600	4358.000
21	AHDR-S 120	37	F	186	415	3490	20	00.000	00.000	00.000	00.000	00.000	00.000	0.995	1.874	55.300	101.600	483.100	15670.000

TDF, tenofovir; FTC, emtricitabine; EFV, efavirenz; CD4, cluster of differentiation 4.

## Discussion

Tenofovir has been the anchor component of most standard first-line ART regimens in low and middle-income countries (LMICs). Taking into account the presence of TDF alone, we observed that 31/77 (40.3%) of the participants had TDF in either their plasma or hair. The concentration of TDF detected in plasma or hair falls within the range of values described in the literature for individuals who are adherent to TDF.^13^ The observed concentrations in hair suggest that individuals may have been on TDF for a significant duration. Noting that about 376 000 of 515 000 PLWH in Limpopo are on ART, our observation that potentially 40% of first-line ART initiates may have had prior exposure to ARVs suggests that up to 55 000 individuals may be at risk of viral resistance and treatment failure.^17^

The study participants had answered ‘no’ to a question on whether they had received ARV medications in any form or programme prior to their current diagnosis and treatment initiation. The detection then of ARV drugs in their tissues requires explanation: some of the women may have previously been on a prevention of mother-to-child transmission (PMTCT) programme,^18^ and this might agree with our observation of the K103N mutation present in women in the pre-treated population. This mutation confers resistance to nevirapine, a non-nucleoside reverse transcriptase inhibitor (NNRTI), and is likely to emerge in women who receive single-dose nevirapine in the PMTCT programme.^19^ Others, men and women, may have previously received ART through a private HIV treatment programme and wished to suppress this information (for whatever reason). Still others may have been ‘silent transfers’ that moved from one public sector health facility to another, without disclosure.^20^ Some participants may have been on treatment for hepatitis B virus (HBV) infection utilising the ARVs, TDF and lamivudine or FTC. Others may have been on pre-exposure or post-exposure prophylaxis in the past. There are also reports on the recreational use of ARVs in *whoonga*/*nyaope* amongst South Africans.^21,22^ Additionally, our research team has anecdotal reports of spouses using the ARV medications of others clandestinely, because of the fear of disclosing their status, as supported by literature.^23^ The question that this observation raises is whether individuals are being initiated onto the standard first-line ART without knowledge of prior exposure to ARV medications? And if this is the case, the sustainability of first-line ART and the expected goals of UTT might potentially be compromised with little impact (observed in Table 5) on patients who are initiated or switched to the new, first-line,^24^ dolutegravir-based ART.

Surprisingly, no significant difference in the prevalence of drug resistance mutations was observed between the participants in whom ARV drugs were detected and those in whom ARV drugs were not detected prior to initiation of treatment. However, mutation distribution amongst both groups was different. Possible explanations for this could be the exposure to TDF for a significant duration of time, or high frequency of minor variants detected using high-sensitivity sequencing technologies,^25^ or it could be that the resistance mutations observed in both groups were largely due to transmitted resistance mutations. Transmitted drug resistance has been shown to be on the rise amongst the pre-treated population in SA with a moderate level of 5–15%. Although resistance is increasing, it is heterogeneous across and within provinces, with the Limpopo province also showing a moderate level.^3^ Of the 31 participants for whom drug resistance data were available at baseline, seven returned for follow-up: four of them had no DRM, whilst three had an NRTI-DRM at the 1% threshold – all of them attained virological suppression at 6 months. Four participants, (one with ARV prior exposure, and three without ARV prior exposure) in whom dual-class NRTI/NNRTI or NRTI/PI resistance weas observed at baseline, could not be further investigated to ascertain the effect of these DRMs on virological suppression at 6 months, since they were lost to follow-up.

A monotonic relationship was observed in the CD4+ cell count and viral copy number in all 21 study participants post-treatment. Several studies have established that VL below the level of detection at post-treatment initiation (HIV RNA < 20 copies/mL – 75 copies/mL, depending on the assay used) indicates optimal viral suppression, and such observations are normal in successfully treated patients and do not predict virological failure.^26^ These studies correlate with our findings, which demonstrated a significant increase in CD4+ cell counts with VL suppression.^27^

The data presented here should be considered in the context of some limitations. Firstly, the small sample size is unlikely to be representative of all who are initiated on ART in the Limpopo province. Secondly, drug resistance data were not available for all 21 participants, who were assessed for adherence based on CD4+ cell count, VL and ARV levels at 6 months post-treatment initiation. So, it was not possible to determine whether or not they harboured resistance mutations going into treatment, and to make the call regarding whether or not the benefits of adherence cancel out the potential impact of these mutations on treatment outcome (i.e. a significant increase in CD4+ cell count, and undetectable VL). Despite these shortcomings, we have objectively shown, by detecting ARV drugs in plasma or hair, that clinicians may be unknowingly recruiting non-drug naïve individuals into HIV treatment programmes on the standard first-line regimen. These results suggest that reporting previous ARV drug exposure accurately is likely to be of benefit in identifying individuals at increased risk of harbouring resistant mutations, and who will require closer follow-up to ensure long-term viral suppression. Disclosure of prior exposure could also assist in the choice of the initial ARV drug regimen, or opting for intensive adherence for these individuals to achieve the desirable treatment outcomes.

## Conclusion

Non-disclosure of previous ART exposure is frequent. Measurement of hair and plasma ARV drugs in PLWH who are NOT yet on ART may identify a group at risk of subsequent treatment failure, and would therefore be a priority group with regard to ART management and follow-up. The presence of resistance related viral mutations and of ARVs in the plasma and hair of ‘ART-naive’ PLWH at the time of ART initiation, suggests the need of surveillance programmes to monitor primary drug-resistance and the establishment of an ‘early-warning’ system to monitor primary HIV-resistance in all areas where ART initiation is provided.

## Appendix 1

### Measurement of CD4+ cell count and viral load

CD4+ cell counts were measured on the BD FACSPresto instrument (Beckton Dickinson) at baseline and after 6 months on ART. Viral loads were determined by using the HIV-1 RNA 3.0 assay (Bayer Healthcare, Leverkusen, Germany). Plasma was prepared by centrifugation of 5 mL whole blood at 4000 rpm for 5 min, and stored in sterile cryovials at –80°C for subsequent experiments.

### Genome sequencing and the determination of viral resistance

Available genetic drug resistance data were obtained by next-generation sequencing on an Illumina MiniSeq platform. Sequences were checked for quality with the Fast quality control (QC) program and trimmed using Geneious Prime version 2020.1.2.

### Antiretroviral drug measurement/quantification in hair and plasma

The analytical standards used in both hair and plasma experiments were *rac*Efavirenz, Emtricitabine and Tenofovir, whilst the internal standards (IS) used were Efavirenz-d5, Lamivudine (3TC) and Tenofovir-d7, for EFV, FTC and TDF, respectively,^28,29,30^ all of which were obtained from Toronto Research Chemicals (Toronto, Canada). 3TC was used as an internal standard instead of FTC, because of their same structural properties; FTC is a fluorinated derivative of 3TC.^31,32^ Negative controls (blank plasma and hair) were prepared from whole blood and hair from volunteers at the Clinical Division of FARMOVS, who had been screened to rule out HIV infection and exposure to antiretrovirals. The chemicals used for the extraction and preparation of calibration standards and QC samples for hair and plasma matrices were acetonitrile, formic acid, methanol, ammonium acetate and trifluoroacetic acid and were procured from MERCK and Honeywell.

### Calibration standards, quality controls and test sample preparation for the plasma matrix

The analytes were isolated from the biological matrix by methanol protein precipitation followed by solid phase extraction. The internal standards (ISTD) were spiked into the extract solution to a concentration of ~1000 ng/mL Efavirenz-d5, ~150 ng/mL Lamivudine and ~500 ng/mL Tenofovir-d7, and the ISTD working solution was added to each sample excluding blank samples.

Calibration standards (STDs) and QC samples were prepared gravimetrically in human K_2_EDTA plasma. A stock solution of each analyte was prepared and used to spike a pool of plasma for the preparation of the STDs. For the preparation of the QCs, a second stock solution of each analyte was prepared and used to spike a pool of plasma. Each pool was then serially diluted with normal blank plasma to attain the desired concentrations and within 1% of target concentrations according to a standard protocol. The target concentrations are assigned as the nominal concentrations. The STDs and QCs were aliquoted and stored at –70°C for subsequent analysis.

The run-containing study samples comprised eight STD levels over the range of 20.00 ng/mL – 2560 ng/mL, with six levels of QC samples extending over this range. Two replicates were included per level for each STD and each QC sample. One blank sample and one zero sample were included in the study sample run.

### Calibration standards, quality controls and test sample preparation for the hair matrix

Approximately 2 mg of hair was weighed in a glass Kimble-tube and a buffer solution consisting of a mixture of trifluoroacetic acid, water and methanol was added. After shaking for 20 h, 1 mL of the extract was evaporated under nitrogen and reconstituted in a solution mixture of methanol and formic acid in water. An ISTD working solution with a concentration of ~400 ng/mL EFV-d5, ~400 ng/mL 3TC and ~50 ng/mL TFV-d7 was prepared, and the working solution was added to each sample excluding blank samples.

Standards and QCs were prepared gravimetrically in a buffer solution. A stock solution of each analyte was prepared and used to spike a pool of buffer for the preparation of the STDs. Quality controls were obtained by preparing a second stock solution of each analyte and using it to spike a pool of buffer. Each pool was then serially diluted with buffer to attain the desired concentrations, acceptable within 1% of the target concentrations. The target concentrations are assigned as the nominal concentrations. The STDs and QCs were aliquoted into individual polypropylene tubes and stored at –20°C until required for the analysis of samples.

### Instrumentation and chromatographic conditions

The LC-MS/MS comprised an autosampler, binary pump, column compartment and sample cooler (AGILENT), with the SCIEX – API4000 and SCIEX – API5500 used for mass spectrometry in the plasma and hair analysis, respectively. The analytical column used for liquid chromatographic separation for both matrices was the Phenomenex^®^ Gemini C18, 150 × 2.00 mm, 5 µm and the mobile phases used (A [100% methanol] and B [0.1% formic acid]) were delivered with a gradient. The autosampler, equipped with a 96-well tray, was used to inject 5 µL of each plasma sample onto the column; 10 µL of each hair sample was injected onto the column.

For each matrix run, the mass spectrometer was set to electrospray ionisation (ESI) in positive ionization mode, for all three analytes. The multiple reaction monitoring (MRM) mode for each analyte and IS was set as follows: *m/z* 316.1 > 244.0 for EFV, *m/z* 321.3 > 172.4 for EFV-d5, *m/z* 248.0 > 130.0 for FTC, *m/z* 230.0 > 112.2 for 3TC, *m/z* 288.1 > 176.2 for TFV, and *m/z* 295.3 > 183.1 for TFV-d7. The retention times for the analytical standards (EFV, FTC, and TFV) and ISTD (EFV-d5, 3TC, and TFV-d7) were 11.69 min, 2.92 min, 2.56 min and 11.69 min, 1.52 min, 2.56 min, respectively.

### Method validation

The qualification run was validated for system suitability (SYS), accuracy, precision, ISTD interference, dilution integrity and carry-over.^29,33^ Accuracy is expressed as the percentage difference between the true nominal concentration and the measured concentration (expressed as % Bias), whilst the precision is expressed as the percentage coefficient of variation (expressed as % CV). The accuracy of the QC samples was within 15% of their respective nominal concentrations; at least 67% of the total number of QC samples in a run and at least one of the two replicates of the QC samples at each concentration level should meet this criterion. The results meet the required acceptance criteria, which indicate that the analytical method is suitable for the simultaneous quantification of TDF, FTC and EFV in human plasma samples over a concentration range of 20.00 ng/mL – 2560 ng/mL based on peak area ratio with the selected calibration curves, as well as in hair samples with concentration ranges being 0.158 ng/mL – 20 ng/mL for EFV, 0.118 ng/mL – 15 ng/mL for FTC and 0.063 ng/mL – 8 ng/mL for TDF, based on peak area ratios with log-log linear calibration curves.
